# Optimum design and performance of a base-isolated structure with tuned mass negative stiffness inerter damper

**DOI:** 10.1038/s41598-023-31482-2

**Published:** 2023-03-27

**Authors:** K. K. Kiran, Mohammed A. Al-Osta, Shamsad Ahmad

**Affiliations:** 1 SJB Institute of Technology, Bangalore, Karnataka India; 2 Department of Civil and Environmental Engineering, King Fahd University of Petroleum and Minerals, Dhahran, 31261 Saudi Arabia; 3 Interdisciplinary Research Center for Construction and Building Materials, King Fahd University of Petroleum and Minerals, Dhahran, 31261 Saudi Arabia

**Keywords:** Seismology, Civil engineering

## Abstract

In order to increase the efficiency of the structures to resist seismic excitation, combinations of inerter, negative stiffness, and tuned mass damper are used. In the present work, the optimum tuning frequency ratio and damping of the tuned mass negative stiffness damper-inerter (TMNSDI) for the base-isolated structure were determined by employing the numerical searching technique under filtered white-noise earthquake excitation and stationary white noise. The energy dissipation index, the absolute acceleration, and the relative displacement of the isolated structure were considered as the optimum parameters, obtained by their maximization. Evaluations of base-isolated structures with and without TMNSDI under non-stationary seismic excitations were investigated. The efficiency of the optimally designed TMNSDI for isolated flexible structures in controlling seismic responses (pulse-type, and real earthquakes) were evaluated in terms of acceleration and displacement. A dynamic system was used for deriving the tuning frequency and tuned mass negative stiffness damper inerter (TMNSDI) for white noise excitation by using explicit formulae of the curve fitting method. The proposed empirical expressions, for design of base-isolated structures with supplementary TMNSDI, showed lesser error. Fragility curve results and story drift ratio indicate reduction in seismic response by 40% and 70% in base-isolated structure using TMNSDI.

## Introduction

The structures are severely damaged when subjected to earthquakes of high intensities resulting in a major loss of life and property. Base isolation of the structures is one of the techniques for reducing the damage of structures due to seismic load. The base isolation works on the principle of adding an element between the ground and superstructure. This element decreases the horizontal stiffness and damping, thus increasing the structure's natural period^1–3^. During the last forty years, base isolation systems have been adopted to reduce damage to the structures due to earthquake load. Hospitals, barracks, firehouses, emergency management, headquarters, etc., are retrofitted structures, and new designs consider the applications of base isolations^4–6^. To protect the structures against earthquake load by virtue of increasing the natural period using base isolation systems, fundamental and energy dissipation capacity methods are important^7^. Globally, base isolation is the most popular method for mitigating structures under seismic load^8,9^. The decoupling behaviour of foundations and superstructure prevents the transfer effects of seismic force by base isolations and prevents the resonance effect of structure^10–12^. Marble graphite slide isolation was proposed for seismic response control^13^. Geotechnical isolation materials used to mitigate the structure under seismic load were investigated by several researchers^14,15^. Composite materials like rubber and concrete were used to isolate the structures exposed to seismic load^16^. In the past, many researchers worked on base isolation systems to make structures resistant to seismic load^17–20^.

Recently, the inerter-based damper method, used for structural vibration control, has gained popularity^21^. The mechanical and electrical network, with force obtained from inerter, was reported by Smith in early 2000^22^. Wind and seismic mitigation of the structure are carried out using a passive control device of a series of damped connections^23–28^. The replacement of a passive (viscous) damper by tuned mass damper (TMD) gives a better structural performance against seismic load by adding a mass of tuned damper that improves story seismic energy by external excitation^29^. An inerter-based isolator achieves higher performance related to traditional TMD by equal inertance-to-mass ratio^30–32^. The single-degree-of-freedom (SDOF) system with an inertia-based damper design method has been developed to control the response of buildings with vibration mitigation^33,34^. Many researchers investigated the mitigation of structures under earthquake load by using inerter damper^35–38^. Supplementary negative stiffness damper is installed to know the performance of a structure against seismic load investigations that many researchers carried out^39–44^. A lower self-weight significantly improves the inertia due to externally applied load resulting from a significant amount of energy. This inertia damper property significantly improves the structure's performance^45^. Some of the researchers carried out studies on inertia dampers in automobiles. These dampers show better performance in vibrations control of automobiles during suspensions^46–49^. Some of the applications of inertia damper on sloshing effects on cylindrical tank response control and optimum design parameter for seismic load are reported in the literature^50,51^. The marine structure response due to seismic load is controlled using an inertia damper^52^. The structure under critical earthquake response is controlled by using an optimum TMD, as reported by Kamgar et al.^53,54^. The optimum TMD parameter under stationary critical excitation was reported by Khatibinia et al.^55^ The seismic design of the modified, tuned liquid damper system can be more effective in reducing the structural responses as investigated by Kamgar et al.^54^. The response of steel moment-resisting frames (SMRF) with TMD under seismic load was investigated by Dhanya et al.^14^. The advantage of frictional TMD compared to conventional TMD is reported by Salimi et al.^56^.

Many researchers have conducted seismic response control of structures utilizing a negative stiffness damper (NSD). The working principle of NSD and analytical and experimental methods pertaining to seismic response control of structure using NSD are reported in the literature^57,58^. Additional evidence on applications of NSD in the mitigation of mechanical vibration equipment and vehicle interruptions can be found in the references^59–62^. A modified NSD as a negative stiffness amplifying damper for controlling the structure's response under seismic and air blast loads is reported by Wang et al., Wang et al. and Kiran et al.^41,42,49^. Different control methods consist of NSD for reducing the response of structures to the seismic load^63,64^. Many researchers reported about response control of the structure system due to seismic load using a NSD^40,61,65–76^. In the case of a base-isolated structure, the superstructure is rigid, whereas the isolation part is flexible to increase the damping effect. An additional supplementary device is attached to mitigate response under external excitation. Tuned mass-damper–inerter (TMDI) is more effective in far-fault (FF) ground motion. A combination of NSD and TMD can control the seismic response of base-isolated structure under near-fault (NF) ground motion.

In the present work, an effort is made to develop an advanced method for hybrid vibration devices for base-isolation of structures under seismic excitation. Therefore, the novelty of this study is summarized below:

(i) Development of fragility curves from nonlinear time history analysis results considering temperature and ageing effects.
(ii) Base isolated structure with supplementary tunned mass negative stiffness damper (TMNSADI) under pulse type excitation.
(iii) Numerical search technique used for finding optimum parameters of TMNSADI.
(iv) Optimization of the parameters of TMNSADI with explicit equation derivation, using curve fitting techniques.
(v) Response reductions of base-isolated structures with supplementer TMNSADI under pulse-type excitation.

## Rigid base-isolated structure with TMNSDI

**Figure 1:**
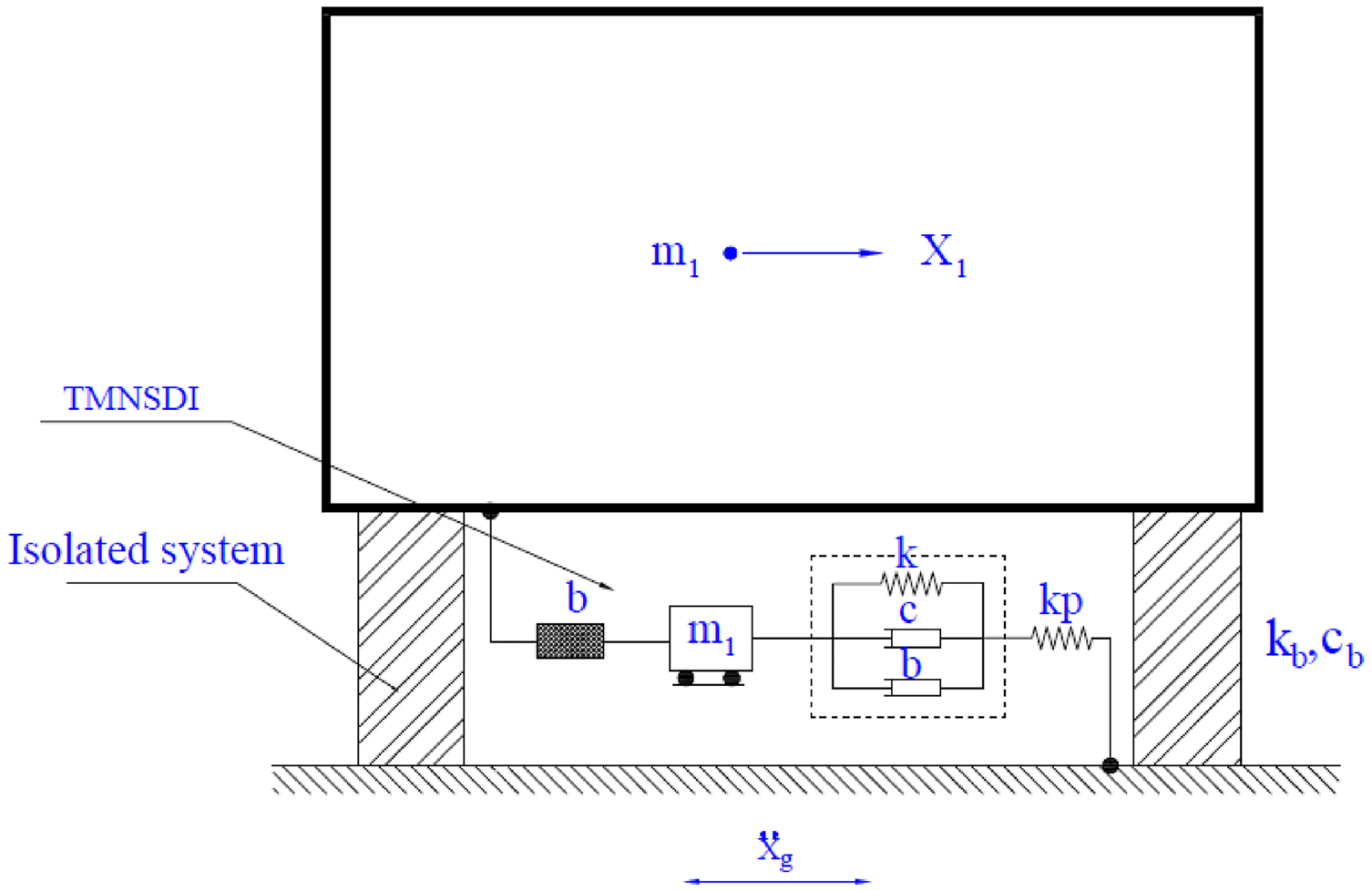
Model of base-isolated structure supplemented with TMNSDI.

In the present study, a Kanai-Tajimi model is considered^77,78^. The model of the isolated structure with supplemental TMNSDI, as shown in Fig. 1, assumes that the effects of torsion in the building are ignored and that the isolated structure has no impact on the adjoining structures^5^. A structural model isolated with supplemental TMNSDI was typically considered, as shown in Fig. 1. Equivalent linear force deformation behaviour of negative stiffness damper was designed for the structure isolation system. The base floor overhead the superstructure mass then isolations is represented as *m*, equivalent stiffness as *k*_*b*_, and damping of isolation as *c*_*b*_. Inertial devices with inertance (*b*), stiffness (*k*_*t*_), and damper (*c*_*t*_) are the parameters that consist of TMNSDI. The relative accelerations among two terminals are assumed to be proportional to the reaction force that happens in inertial devices of the movement. TMNSDI consists of an auxiliary mass (*m*_*t*_).

The base isolation scheme is considered for two-parameter of viz T_b_ and ξ_b_, as given below^79^:

1a $$ {\text{T}}_{{\text{b}}} = \frac{{{2}\uppi }}{{{\upomega }_{{\text{b}}} }} $$

1b $$ {\upomega }_{{\text{b}}} = \sqrt {\frac{{{\text{k}}_{{\text{b}}} }}{{\text{m}}}} $$

1c $$ 2\xi_{{\text{b}}} \omega_{{\text{b}}} = \frac{{{\text{c}}_{{\text{b}}} }}{{\text{m}}} $$

The auxiliary and inertial masses of the TMNSDI are defined as:

2a $$ {\upmu }_{{\text{t}}} { = }\frac{{{\text{m}}_{{\text{t}}} }}{{\text{m}}},\quad {\upmu }_{{\text{b}}} { = }\frac{{\text{b}}}{{\text{m}}} $$

2b $$ {\upmu } = {\upmu }_{{\text{t}}} + {\upmu }_{{\text{b}}} { = }\frac{{{\text{m}}_{{\text{t}}} + {\text{b}}}}{{{1}{\text{.5m}}}} $$

TMNSDI base-isolated structure of stiffness and damping parameter is defined as:

3a $$ \xi_{t} = \frac{{{\text{c}}_{{\text{t}}} }}{{{2}{\text{.5(m}}_{{\text{t}}} + {\text{b)w}}_{{\text{t}}} }} $$

3b $$ \omega_{t} = \sqrt {\frac{{k_{t} }}{{{\text{m}}_{{\text{t}}} + {\text{b}}}}} $$

3c $$ f = \frac{{\omega_{t} }}{{\omega_{b} }} $$

where the ratio of damping is $\xi_{t}$, and the ratio of tunning frequency is *f* for TMNSDI of the base-isolated structure. The $\mu$, $\xi_{t}$ and *f* are focal parameters for TMNSDI of base-isolated structure. In the present work, $\mu_{t} = 0.01$ is considered for all parametric studies because TMDI is more real for inferior values of ${\upmu }_{{\text{t}}}$ and advanced values of ${\upmu }$^80^.

The governing equations of motion can be expressed as:

4 $$ \left[ {\begin{array}{*{20}c} {\text{m}} & {\text{o}} \\ {\text{o}} & {{\text{m}}_{{\text{t}}} + {\text{b}}} \\ \end{array} } \right]\left\{ {\begin{array}{*{20}c} {{\ddot{\text{x}}}} \\ {\mathop {{\text{x}}_{{\text{t}}} }\limits^{..} } \\ \end{array} } \right\}{ + }\left[ {\begin{array}{*{20}c} {{\text{c}}_{{\text{b}}} + {\text{c}}_{{\text{t}}} } & { - {\text{c}}_{{\text{t}}} } \\ { - {\text{c}}_{{\text{t}}} } & {{\text{c}}_{{\text{t}}} } \\ \end{array} } \right]\left\{ {\begin{array}{*{20}c} {{\dot{\text{x}}}} \\ {\mathop {{\text{x}}_{{\text{t}}} }\limits^{.} } \\ \end{array} } \right\}{ + }\left[ {\begin{array}{*{20}c} {{\text{k}}_{{\text{b}}} + {\text{k}}_{{\text{t}}} } & { - {\text{k}}_{{\text{t}}} } \\ { - {\text{k}}_{{\text{t}}} } & {{\text{k}}_{{\text{t}}} } \\ \end{array} } \right]\left\{ {\begin{array}{*{20}c} {\text{x}} \\ {{\text{x}}_{{\text{t}}} } \\ \end{array} } \right\}{ = } - \left\{ {\begin{array}{*{20}c} {\text{m}} \\ {{\text{m}}_{{\text{t}}} } \\ \end{array} } \right\}\left( {{\ddot{\text{x}}}_{{\text{g}}} } \right) $$

in which *x* and *x*_*t*_ are the displacements comparative to the ground of the isolated structure with TMNSDI. The ground accelerations are represented as $\ddot{x}_{g}$. The dot over the symbol represents variation with respect to time *t.*

### Response under Gaussian white noise excitation

The state space format of Eq. (4) can be expressed as^81^:

5 $$ {\dot{\text{Z}}} = {\text{SZ}} + {\text{U}} $$

where *Z*, *S*, and *U* are the state vector, and the excitation of the input vector are shown as

6 $$ {\text{Z}} = \left\{ {\begin{array}{*{20}c} {{\text{x}}_{{\text{s}}} } & {\mathop {{\text{x}}_{{\text{s}}} }\limits^{.} } & {{\text{x}}_{{\text{t}}} } & {\mathop {{\text{x}}_{{\text{t}}} }\limits^{.} } \\ \end{array} } \right\}^{{\text{T}}} $$

7 $$ {\text{U}} = \left\{ {\begin{array}{*{20}c} {0} & {0} & {1} & {\frac{{{\text{m}}_{{\text{t}}} }}{{{\text{m}}{}_{{\text{t}}}{\text{ + b}}}}} \\ \end{array} } \right\}^{{\text{T}}} \mathop {\text{x}}\limits^{..}_{{\text{g}}} {\text{ = B}}\mathop {\text{x}}\limits^{..}_{{\text{g}}} $$

The response of vector Markov process *Z* satisfies covariance matrix Y, corresponding with the following equations^82^.

8 $$ \mathop {\text{Y}}\limits^{.} = {\text{SY}}^{{\text{T}}} + {\text{YS}}^{{\text{T}}} + {\text{P}} $$

where *Y* transpose matrix is represented as *Y*^*T*^.

The Y and P are the element matrix, as given below:

9 $$ {\text{Y}}_{{{\text{ij}}}} = {\text{E}}\left[ {{\text{Z}}_{{\text{i}}} {\text{Z}}_{{\text{j}}} } \right]\quad {\text{and}}\quad {\text{P}}_{{{\text{ij}}}} = {\text{E}}\left[ {{\text{U}}_{{\text{i}}} {\text{U}}_{{\text{j}}} } \right] $$

where *E* is the expectation operator and *i*^*th*^ and *j*^*th*^ elements of the vector are represented by *Z* and *U* are *Z*_*i*_ and *U*_*i,*_ respectively.

Considering the power spectral density function (PSDF) as *S*_*o*_ and Gaussian zero-mean white noise random process as the input of stationary earthquake of acceleration $\ddot{x}_{g}$, the *P* matrix is expressed as^82^:

10 $$ {\text{P}} = {2}\uppi {\text{S}}_{{\text{o}}} {\text{BB}}^{{\text{T}}} $$

Base isolated structures with supplemental TMNSDI under stationary earthquake response are the matrix of null as $\dot{Y}$, given by Eq. (8). The relative displacement means square (${\upsigma }_{{\text{x}}}^{{2}}$) and acceleration absolute ($\ddot{x}_{a} = \mathop {x_{s} }\limits^{..} + \ddot{x}_{g}$) as (${\upsigma }_{{^{{\mathop {{\text{x}}_{{\text{a}}} }\limits^{..} }} }}^{{2}}$) for structure with isolated are obtained by the elements of the Y matrix. The base-isolated structure response without TMNSDI is normalized as follows:

11 $$ {\tilde{\sigma }}_{{{\text{x}}_{{\text{s}}} }}^{{2}} { = }\frac{{{\upsigma }_{{{\text{x}}_{{\text{s}}} }}^{{2}} }}{{{\upsigma }_{{{\text{x}}_{{\text{s}}} {,0}}}^{{2}} }}\quad {\text{and}}\quad {\tilde{\sigma }}_{{\ddot{x}_{{\text{a}}} }}^{{2}} { = }\frac{{{\upsigma }_{{\ddot{x}_{{\text{a}}} }}^{{2}} }}{{{\upsigma }_{{\ddot{x}_{{\text{a}}} {,0}}}^{{2}} }} $$

12 $$\upsigma _{{\text{x,0}}}^{{2}} = \frac{{\uppi {\text{S}}_{{0}} }}{{{2}\upxi _{{\text{s}}} {\upomega }_{{\text{s}}}^{{3}} }}\quad {\text{and}}\quad\upsigma _{{{\ddot{\text{x}}}_{{\text{a}}} {,0}}}^{{2}} =\uppi {\text{S}}_{{0}}\upomega _{{\text{s}}} \left( {\frac{{1}}{{{2}\upxi _{{\text{s}}} }} + {2}\upxi _{{\text{s}}} } \right) $$

The investigations for energy criterion are carried out based on the accelerations, displacement, and base isolation structure. The performance of tuned inerter damper (TID) is carried out using the energy criterion given by^81^. The TMNSDI plays a vital role in dispersing energy for the earthquake fervor induced by the total energy input. The expressions for the energy dissipation index (EDI) are shown below:

13 $$ {\text{EDI}} = \frac{{{\text{E}}\left[ {{\Delta E}_{{\text{T}}} } \right]}}{{{\text{E}}\left[ {{\Delta E}_{{\text{s}}} } \right] + {\text{E}}\left[ {{\Delta E}_{{\text{T}}} } \right]}} $$

where dissipated energy increment for negative stiffness of tunned inertia damper and negative stiffness damping of base-isolated structure are ${\text{E}}\left[ {{\Delta E}_{{\text{s}}} } \right]$ and ${\text{E}}\left[ {{\Delta E}_{{\text{T}}} } \right]$, respectively. Limits of the EDI vary between zero and unity, and the value increases with an increase in dissipating energy of TMNSDI.

The element of the Y matrix with respect to EDI is given below:

14 $$ {\text{EDI}} = \frac{{{\text{c}}_{{\text{t}}} {\upsigma }_{{{\dot{\text{x}}}_{{\text{r}}} }}^{{2}} }}{{{\text{c}}_{{\text{t}}} {\upsigma }_{{\mathop {{\text{x}}_{{\text{s}}} }\limits^{.} }}^{{2}} + {\text{c}}_{{\text{t}}} {\upsigma }_{{{\dot{\text{x}}}_{{\text{r}}} }}^{{2}} }} $$

where ${\upsigma }_{{\mathop {{\text{x}}_{{\text{s}}} }\limits^{.} }}^{{2}}$ and ${\upsigma }_{{{\dot{\text{x}}}_{{\text{r}}} }}^{{2}}$ are velocity variance for $\mathop {{\text{x}}_{{\text{s}}} }\limits^{.}$ and ${\dot{\text{x}}}_{{\text{r}}} = {\dot{\text{x}}}_{{\text{t}}} - \mathop {{\text{x}}_{{\text{s}}} }\limits^{.}$, respectively.

## Proposed control system characteristics

### Limitation

In the proposed control system, it is not possible to control the response of the tall structure, which is frequently exposed to wind load. Also, structures exposed to multi-hazards such as combinations of several loads such as blast load, the seismic load acting in bi-directional, torsional effect, volcanic load, etc. It is used in low-rise structures, base-isolated structures (maximum five stories), and SDOF systems under seismic load.

### Hypothesis and construction

The proposed hypothesis of the control system that is tuned mass negative stiffness inerter damper are as follows: (i) base isolated structure in each superstructure's floor diaphragm is assumed to be rigid enough in comparison to the columns, (ii) one of earthquake acceleration's horizontal component is considered to be the isolated structural system, and any interaction effects provided by additional vertical and orthogonal horizontal components are simply neglected, and (iii) the floor's lateral stiffness is assumed to be linear with *k*_*i*_ representing the stiffness of *i*^*th*^ floor.

The proposed control system construction procedure for a negative stiffness damper is adopted from the work reported by^83^, in which passive control device such as negative stiffness damper was considered. In the case of tuned mass damper inerter, the parameter considered was similar to that obtained experimentally by^84^.

## Optimum parameters of TMNSDI

### Under Gaussian white noise excitation

15 $$ {\text{Minimizing}}\;\tilde{\sigma }_{x}^{2} \;{\text{subject to}}\;{\upxi }_{{\text{t}}} \in {\Omega }_{{\upxi }} ,{\text{f}} \in {\Omega }_{{\text{f}}} ,\quad {\text{f}} \in {\Omega }_{{\text{f}}} $$

16 $$ {\text{Minimizing}}\;\tilde{\sigma }_{{\mathop {x_{a} }\limits^{..} }}^{2} \;{\text{subject to}}\;{\upxi }_{{\text{t}}} \in {\Omega }_{{\upxi }} ,{\text{f}} \in {\Omega }_{{\text{f}}} ,\quad {\text{f}} \in {\Omega }_{{\text{f}}} $$

17 $$ {\text{Maximizing EDI subject to}}\;{\upxi }_{{\text{t}}} \in {\Omega }_{{\upxi }} ,{\text{f}} \in {\Omega }_{{\text{f}}} ,\quad {\text{f}} \in {\Omega }_{{\text{f}}} $$

where positive orthants for the associated variables for ${\upxi }_{{\text{t}}}$ and f are ${\Omega }_{{\upxi }}$ and ${\Omega }_{{\text{f}}}$ feasible regions, respectively. $0 < {\Omega }_{{\upxi }} < {1}$ and $0 < {\Omega }_{{\text{f}}} < {2}$ are considered feasible regions, respectively. For obtaining an increment of 10^–5^ to reach an automatic new search algorithm ${\upxi }_{{\text{t}}}$, and positive orthants of corresponding variables are expressed as f is considered.

The results of TMNSDI for optimized parameters of minimization of $\tilde{\sigma }_{x}^{2}$ are shown in Table 1. It is observed from Table 1 that the increase in the values of $\upmu$ and ${\upxi }_{{\text{t}}}^{{{\text{opt}}}}$ results in the decrease of ${\text{f}}^{{{\text{opt}}}}$. Further, the increase in value of ${\upmu }$ caused reduction in the values of $\tilde{\sigma }_{{x_{s} }}^{2}$ and $\tilde{\sigma }_{{\mathop {x_{a} }\limits^{..} }}^{2}$.The mass ratio also plays a vital role in making the inertance more effective. For the value of the mass ratio of 0.4, deviations of the ${\upxi }_{{\text{t}}}^{{{\text{opt}}}}$, ${\text{f}}^{{{\text{opt}}}}$, $\tilde{\sigma }_{{x_{s} }}^{2}$ and $\tilde{\sigma }_{{\mathop {x_{a} }\limits^{..} }}^{2}$ occur. To find optimal TMNSDI parameters with ${\upxi }_{{\text{t}}}$ and f in their ranges with an increment of 10^–4^, a numerical search technique was used with the help of MATLAB script. For each value of ${\upmu }$, different values of the major possible damping system of the structure (${\upxi }_{{\text{s}}}$) were considered as 0, 0.02, 0.05, 0.075, and 0.1. It is observed that for the given value of ${\upmu }$, ${\upxi }_{{\text{s}}}$ increases due to the effect of the negative stiffness damper on the inerter of the base isolation structure and ${\text{f}}^{{{\text{opt}}}}$ decreases due to the effect of the tuned inerter damper on the base-isolated structure and ${\upxi }_{{\text{t}}}^{{{\text{opt}}}}$ approximately remains the same. For the values of ${\upmu } > 0.5$, both ${\text{f}}^{{{\text{opt}}}}$ and ${\upxi }_{{\text{s}}}$ values increase. The higher inertance mass ratio and lower structural damping are found to be more effective at a higher optimum value of ${\tilde{\sigma }}_{{{\text{x}}_{{\text{s}}} }}^{{2}}$. Table 2 shows the results of the minimization $\tilde{\sigma }_{{\mathop {x_{a} }\limits^{..} }}^{2}$. A similar trend of the values of ${\text{f}}^{{{\text{opt}}}}$ and ${\upxi }_{{\text{t}}}^{{{\text{opt}}}}$ was observed as the ${\upmu }$ value increased. The minimization of acceleration response is lesser than the minimization of displacement response for the ${\text{f}}^{{{\text{opt}}}}$ and ${\upxi }_{{\text{t}}}^{{{\text{opt}}}}$ parameters. The optimum results obtained by maximization of EDI are illustrated in Table 3. EDI maximization results also show a trend similar to that obtained by minimization of $\tilde{\sigma }_{x}^{2}$ and $\tilde{\sigma }_{{\mathop {x_{a} }\limits^{..} }}^{2}$.

**Table 1:** TMNSDI of optimum damper achieved with minimization of $\tilde{\sigma }_{x}^{2}$.

${\upmu }$	${\upxi }_{{\text{s}}} = 0$		${\upxi }_{{\text{s}}} = 0.02$			${\upxi }_{{\text{s}}} = 0.05$			${\upxi }_{{\text{s}}} = 0.075$			${\upxi }_{{\text{s}}} = 0.1$		
	${\text{f}}^{{{\text{opt}}}}$	${\upxi }_{{\text{t}}}^{{{\text{opt}}}}$	${\text{f}}^{{{\text{opt}}}}$	${\upxi }_{{\text{t}}}^{{{\text{opt}}}}$	${\tilde{\sigma }}_{{{\text{x}}_{{\text{s}}} }}^{{2}} {\text{,opt}}$	${\text{f}}^{{{\text{opt}}}}$	${\upxi }_{{\text{t}}}^{{{\text{opt}}}}$	${\tilde{\sigma }}_{{{\text{x}}_{{\text{s}}} }}^{{2}} {\text{,opt}}$	${\text{f}}^{{{\text{opt}}}}$	${\upxi }_{{\text{t}}}^{{{\text{opt}}}}$	${\tilde{\sigma }}_{{{\text{x}}_{{\text{s}}} }}^{{2}} {\text{,opt}}$	${\text{f}}^{{{\text{opt}}}}$	${\upxi }_{{\text{t}}}^{{{\text{opt}}}}$	${\tilde{\sigma }}_{{{\text{x}}_{{\text{s}}} }}^{{2}}$
0.01	1.3278	0.0498	1.3147	0.0498	0.6388	1.2958	0.0498	1.3768	1.2807	0.0498	1.8634	1.266	0.0498	2.2577
0.02	1.3224	0.0701	1.3093	0.0701	0.4687	1.2905	0.0701	1.0409	1.2754	0.0701	1.4414	1.2608	0.0701	1.787
0.03	1.317	0.0855	1.304	0.0855	0.3898	1.2852	0.0855	0.8779	1.2702	0.0855	1.2284	1.2557	0.0855	1.5383
0.04	1.3116	0.0983	1.2987	0.0983	0.3416	1.28	0.0983	0.7761	1.265	0.0983	1.0928	1.2506	0.0983	1.3768
0.05	1.3064	0.1094	1.2935	0.1094	0.3081	1.2749	0.1094	0.7044	1.26	0.1094	0.9963	1.2456	0.1094	1.2605
0.075	1.2934	0.1327	1.2807	0.1327	0.2551	1.2623	0.1327	0.5893	1.2475	0.1327	0.8394	1.2332	0.1327	1.0691
0.1	1.2809	0.1517	1.2682	0.1517	0.223	1.25	0.1517	0.5183	1.2354	0.1517	0.7415	1.2213	0.1517	0.9483
0.125	1.2686	0.1679	1.2561	0.1679	0.2007	1.2381	0.1679	0.4687	1.2236	0.1679	0.6727	1.2096	0.1679	0.8628
0.15	1.2568	0.1821	1.2444	0.1821	0.1842	1.2265	0.1821	0.4315	1.2121	0.1821	0.6208	1.1983	0.1821	0.798
0.175	1.2452	0.1949	1.2329	0.1949	0.1712	1.2152	0.1949	0.4023	1.201	0.1949	0.5798	1.1872	0.1949	0.7466
0.2	1.2339	0.2064	1.2217	0.2064	0.1607	1.2042	0.2064	0.3784	1.1901	0.2064	0.5463	1.1765	0.2064	0.7044
0.225	1.2229	0.217	1.2109	0.217	0.1519	1.1934	0.217	0.3585	1.1795	0.217	0.5183	1.166	0.217	0.669
0.25	1.2122	0.2267	1.2002	0.2267	0.1445	1.183	0.2267	0.3416	1.1691	0.2267	0.4943	1.1558	0.2267	0.6388
0.275	1.2017	0.2357	1.1899	0.2357	0.1381	1.1728	0.2357	0.3269	1.1591	0.2357	0.4735	1.1458	0.2357	0.6124
0.3	1.1915	0.2441	1.1798	0.2441	0.1325	1.1628	0.2441	0.314	1.1492	0.2441	0.4552	1.1361	0.2441	0.5893
0.325	1.1816	0.2519	1.1699	0.2519	0.1275	1.1531	0.2519	0.3025	1.1396	0.2519	0.439	1.1266	0.2519	0.5687
0.35	1.1719	0.2593	1.1603	0.2593	0.123	1.1436	0.2593	0.2923	1.1302	0.2593	0.4245	1.1173	0.2593	0.5502
0.375	1.1624	0.2662	1.1509	0.2662	0.119	1.1344	0.2662	0.2831	1.1211	0.2662	0.4113	1.1083	0.2662	0.5335
0.4	1.1531	0.2728	1.1417	0.2728	0.1154	1.1253	0.2728	0.2747	1.1121	0.2728	0.3994	1.0994	0.2728	0.5183
0.425	1.144	0.279	1.1328	0.279	0.1121	1.1165	0.279	0.2671	1.1034	0.279	0.3885	1.0908	0.279	0.5043
0.45	1.1352	0.2849	1.124	0.2849	0.1091	1.1078	0.2849	0.26	1.0949	0.2849	0.3784	1.0823	0.2849	0.4915
0.475	1.1265	0.2905	1.1154	0.2905	0.1063	1.0994	0.2905	0.2535	1.0865	0.2905	0.3692	1.0741	0.2905	0.4797
0.5	1.118	0.2958	1.107	0.2958	0.1037	1.0911	0.2958	0.2475	1.0783	0.2958	0.3606	1.066	0.2958	0.4687
0.5	1.118	0.2958	1.107	0.2958	0.1037	1.0911	0.2958	0.2475	1.0783	0.2958	0.3606	1.066	0.2958	0.4687
0.6	1.0859	0.3149	1.0752	0.3149	0.095	1.0597	0.3149	0.2273	1.0473	0.3149	0.3315	1.0353	0.3149	0.4315
0.7	1.0563	0.3311	1.0459	0.3311	0.0882	1.0308	0.3311	0.2114	1.0188	0.3311	0.3087	1.0071	0.3311	0.4023
0.8	1.0289	0.345	1.0187	0.345	0.0827	1.0041	0.345	0.1985	0.9923	0.345	0.2902	0.981	0.345	0.3784
0.9	1.0035	0.3572	0.9936	0.3572	0.0781	0.9793	0.3572	0.1877	0.9678	0.3572	0.2747	0.9568	0.3572	0.3585
1	0.9798	0.368	0.9701	0.368	0.0742	0.9562	0.368	0.1786	0.945	0.368	0.2615	0.9342	0.368	0.3416

**Table 2:** TMNSDI of optimum damper achieved with minimization of $\tilde{\sigma }_{{\mathop {x_{a} }\limits^{..} }}^{2}$.

${\upmu }$	${\upxi }_{{\text{s}}} = 0$		${\upxi }_{{\text{s}}} = 0.02$			${\upxi }_{{\text{s}}} = 0.05$			${\upxi }_{{\text{s}}} = 0.075$			${\upxi }_{{\text{s}}} = 0.1$		
	${\text{f}}^{{{\text{opt}}}}$	${\upxi }_{{\text{t}}}^{{{\text{opt}}}}$	${\text{f}}^{{{\text{opt}}}}$	${\upxi }_{{\text{t}}}^{{{\text{opt}}}}$	${\tilde{\sigma }}_{{_{{\mathop {x_{a} }\limits^{..} }} opt}}^{{2}}$	${\text{f}}^{{{\text{opt}}}}$	${\upxi }_{{\text{t}}}^{{{\text{opt}}}}$	${\tilde{\sigma }}_{{_{{\mathop {x_{a} }\limits^{..} }} opt}}^{{2}}$	${\text{f}}^{{{\text{opt}}}}$	${\upxi }_{{\text{t}}}^{{{\text{opt}}}}$	${\tilde{\sigma }}_{{_{{\mathop {x_{a} }\limits^{..} }} opt}}^{{2}}$	${\text{f}}^{{{\text{opt}}}}$	${\upxi }_{{\text{t}}}^{{{\text{opt}}}}$	${\tilde{\sigma }}_{{_{{\mathop {x_{a} }\limits^{..} }} opt}}^{{2}}$
0.01	0.9967	0.0615	0.9995	0.0628	0.3907	1.0078	0.0646	0.6684	1.0171	0.0662	0.7183	1.0282	0.0677	0.641
0.02	0.9933	0.0874	0.9961	0.0892	0.2997	1.0044	0.0918	0.5645	1.0137	0.094	0.6747	1.0247	0.0962	0.7057
0.03	0.9899	0.1076	0.9927	0.1097	0.2544	1.001	0.1129	0.4991	1.0103	0.1156	0.6202	1.0213	0.1183	0.6812
0.04	0.9866	0.1247	0.9894	0.1272	0.2259	0.9976	0.131	0.4538	1.0068	0.1341	0.5762	1.0178	0.1372	0.6491
0.05	0.9832	0.14	0.986	0.1428	0.2056	0.9942	0.147	0.4199	1.0034	0.1505	0.5408	1.0143	0.154	0.619
0.075	0.9747	0.173	0.9774	0.1765	0.1728	0.9856	0.1817	0.3621	0.9947	0.186	0.4765	1.0055	0.1904	0.5583
0.1	0.9661	0.2015	0.9688	0.2055	0.1524	0.9769	0.2115	0.3244	0.9859	0.2166	0.4323	0.9966	0.2216	0.5132
0.125	0.9574	0.2269	0.9601	0.2314	0.1381	0.9681	0.2382	0.2972	0.9771	0.2439	0.3994	0.9877	0.2496	0.4784
0.15	0.9487	0.2501	0.9514	0.2551	0.1273	0.9593	0.2626	0.2763	0.9681	0.2689	0.3737	0.9786	0.2751	0.4504
0.175	0.9399	0.2717	0.9425	0.2771	0.1188	0.9503	0.2853	0.2596	0.9591	0.2921	0.3528	0.9695	0.2989	0.4273
0.2	0.9309	0.2919	0.9336	0.2978	0.1119	0.9413	0.3065	0.2457	0.95	0.3138	0.3353	0.9603	0.3211	0.4078
0.225	0.922	0.311	0.9245	0.3172	0.1061	0.9322	0.3266	0.234	0.9408	0.3344	0.3204	0.9509	0.3421	0.391
0.25	0.9129	0.3291	0.9154	0.3357	0.1012	0.923	0.3456	0.224	0.9314	0.3538	0.3075	0.9415	0.3621	0.3763
0.275	0.9037	0.3464	0.9062	0.3533	0.0969	0.9137	0.3637	0.2152	0.922	0.3724	0.2962	0.9319	0.381	0.3632
0.3	0.8944	0.3629	0.8969	0.3701	0.0931	0.9042	0.381	0.2074	0.9124	0.3901	0.2861	0.9221	0.3992	0.3516
0.325	0.8851	0.3787	0.8875	0.3863	0.0898	0.8947	0.3976	0.2005	0.9027	0.4071	0.2771	0.9123	0.4166	0.3412
0.35	0.8756	0.3939	0.878	0.4018	0.0868	0.885	0.4136	0.1943	0.8929	0.4234	0.2689	0.9022	0.4333	0.3317
0.375	0.866	0.4085	0.8684	0.4167	0.0841	0.8752	0.4289	0.1886	0.8829	0.4391	0.2615	0.8921	0.4493	0.323
0.4	0.8563	0.4226	0.8586	0.431	0.0816	0.8653	0.4437	0.1835	0.8728	0.4543	0.2547	0.8817	0.4648	0.315
0.425	0.8466	0.4362	0.8488	0.4449	0.0794	0.8553	0.458	0.1787	0.8625	0.4689	0.2485	0.8711	0.4798	0.3077
0.45	0.8367	0.4493	0.8388	0.4583	0.0773	0.8451	0.4718	0.1744	0.8521	0.483	0.2427	0.8604	0.4942	0.3009
0.475	0.8266	0.462	0.8287	0.4713	0.0754	0.8347	0.4851	0.1703	0.8415	0.4967	0.2374	0.8495	0.5082	0.2945
0.5	0.8165	0.4743	0.8184	0.4838	0.0736	0.8242	0.4981	0.1666	0.8306	0.5099	0.2324	0.8383	0.5218	0.2886
0.5	0.8165	0.4743	0.8184	0.4838	0.0736	0.8242	0.4981	0.1666	0.8306	0.5099	0.2324	0.8383	0.5218	0.2886
0.6	0.7746	0.52	0.7761	0.5304	0.0677	0.7805	0.546	0.1538	0.7854	0.559	0.2153	0.7912	0.572	0.2683
0.7	0.7303	0.5608	0.7311	0.5721	0.063	0.7336	0.5889	0.1438	0.7363	0.6029	0.2018	0.7395	0.6169	0.2521
0.8	0.6831	0.5976	0.6831	0.6096	0.0592	0.6829	0.6275	0.1355	0.6828	0.6424	0.1906	0.6826	0.6574	0.2386
0.9	0.6325	0.631	0.6313	0.6436	0.056	0.6279	0.6625	0.1286	0.6241	0.6783	0.1812	0.6197	0.6941	0.2273
1	0.5774	0.6614	0.5749	0.6747	0.0533	0.5677	0.6945	0.1227	0.5596	0.711	0.1732	0.55	0.7276	0.2175

**Table 3:** TMNSDI of optimum damper achieved with maximization of EDI.

${\upmu }$	${\upxi }_{{\text{s}}} = 0.02$			${\upxi }_{{\text{s}}} = 0.05$			${\upxi }_{{\text{s}}} = 0.075$			${\upxi }_{{\text{s}}} = 0.1$		
	${\text{f}}^{{{\text{opt}}}}$	${\upxi }_{{\text{t}}}^{{{\text{opt}}}}$	${\tilde{\sigma }}_{{_{{\mathop {x_{a} }\limits^{..} }} opt}}^{{2}}$	${\text{f}}^{{{\text{opt}}}}$	${\upxi }_{{\text{t}}}^{{{\text{opt}}}}$	${\tilde{\sigma }}_{{_{{\mathop {x_{a} }\limits^{..} }} opt}}^{{2}}$	${\text{f}}^{{{\text{opt}}}}$	${\upxi }_{{\text{t}}}^{{{\text{opt}}}}$	${\tilde{\sigma }}_{{_{{\mathop {x_{a} }\limits^{..} }} opt}}^{{2}}$	${\text{f}}^{{{\text{opt}}}}$	${\upxi }_{{\text{t}}}^{{{\text{opt}}}}$	${\tilde{\sigma }}_{{_{{\mathop {x_{a} }\limits^{..} }} opt}}^{{2}}$
0.01	0.9952	0.0509	0.4255	0.9958	0.0524	− 0.0156	0.9967	0.0537	− 0.2799	0.998	0.0549	− 0.5001
0.02	0.9903	0.0719	0.5049	0.9912	0.074	0.0644	0.9925	0.0758	− 0.2047	0.9943	0.0775	− 0.4292
0.03	0.9855	0.0879	0.5593	0.9866	0.0905	0.1291	0.9882	0.0927	− 0.1403	0.9904	0.0948	− 0.3664
0.04	0.9808	0.1014	0.5997	0.9821	0.1044	0.1828	0.9839	0.1068	− 0.0843	0.9865	0.1093	− 0.3104
0.05	0.9762	0.1132	0.6311	0.9775	0.1165	0.2285	0.9796	0.1193	− 0.0351	0.9824	0.1221	− 0.2599
0.075	0.9648	0.1382	0.6871	0.9665	0.1423	0.318	0.9689	0.1457	0.0661	0.9724	0.149	− 0.1528
0.1	0.9538	0.1592	0.7248	0.9557	0.1638	0.3845	0.9586	0.1678	0.1453	0.9625	0.1717	− 0.066
0.125	0.9432	0.1776	0.7524	0.9453	0.1828	0.4366	0.9484	0.1871	0.2094	0.9528	0.1915	0.0062
0.15	0.9329	0.1942	0.7738	0.9352	0.1999	0.4787	0.9386	0.2046	0.2628	0.9433	0.2094	0.0675
0.175	0.923	0.2094	0.7911	0.9254	0.2156	0.5138	0.929	0.2207	0.3081	0.9341	0.2258	0.1204
0.2	0.9134	0.2236	0.8054	0.9159	0.2302	0.5436	0.9198	0.2357	0.3472	0.9251	0.2412	0.1667
0.225	0.904	0.2371	0.8175	0.9067	0.244	0.5693	0.9107	0.2499	0.3814	0.9164	0.2557	0.2076
0.25	0.895	0.2498	0.8279	0.8978	0.2572	0.5918	0.902	0.2633	0.4117	0.9078	0.2694	0.2441
0.275	0.8862	0.2621	0.8369	0.8891	0.2698	0.6116	0.8935	0.2762	0.4386	0.8995	0.2827	0.2769
0.3	0.8776	0.2739	0.845	0.8807	0.282	0.6294	0.8852	0.2887	0.4629	0.8915	0.2954	0.3066
0.325	0.8693	0.2854	0.8521	0.8725	0.2938	0.6453	0.8771	0.3008	0.4849	0.8836	0.3078	0.3337
0.35	0.8613	0.2966	0.8585	0.8645	0.3053	0.6598	0.8693	0.3126	0.5049	0.8759	0.3198	0.3585
0.375	0.8534	0.3075	0.8643	0.8567	0.3166	0.673	0.8616	0.3241	0.5233	0.8685	0.3317	0.3813
0.4	0.8458	0.3183	0.8696	0.8492	0.3277	0.685	0.8542	0.3355	0.5401	0.8612	0.3433	0.4024
0.425	0.8384	0.329	0.8744	0.8418	0.3387	0.6961	0.8469	0.3467	0.5557	0.8541	0.3548	0.4219
0.45	0.8311	0.3396	0.8789	0.8346	0.3496	0.7064	0.8399	0.3579	0.5702	0.8472	0.3662	0.4401
0.475	0.8241	0.3501	0.883	0.8276	0.3604	0.7159	0.833	0.369	0.5837	0.8404	0.3776	0.4571
0.5	0.8172	0.3606	0.8868	0.8208	0.3712	0.7248	0.8262	0.3801	0.5962	0.8338	0.3889	0.473
0.5	0.8172	0.3606	0.8868	0.8208	0.3712	0.7248	0.8262	0.3801	0.5962	0.8338	0.3889	0.473
0.6	0.7913	0.4032	0.8996	0.7952	0.415	0.7549	0.8009	0.4249	0.6392	0.8089	0.4348	0.5275
0.7	0.7677	0.4481	0.9096	0.7718	0.4613	0.7786	0.7778	0.4723	0.6733	0.7862	0.4833	0.5713
0.8	0.7462	0.4977	0.9177	0.7504	0.5123	0.7979	0.7566	0.5245	0.7012	0.7654	0.5367	0.6072
0.9	0.7263	0.555	0.9244	0.7306	0.5713	0.8139	0.7371	0.5849	0.7245	0.7461	0.5985	0.6373
1	0.708	0.6246	0.93	0.7124	0.643	0.8275	0.719	0.6583	0.7443	0.7283	0.6736	0.6629

### Optimum TMNSDI parameters of closed form of solution

Numerical search techniques are adopted to select the optimum values of TMNSDI parameters. The optimum values of TMNSDI parameters were obtained using explicit mathematical expressions by utilizing the curve fitting technique. The role of $\mu$ for the specified isolated damping ratio (${\upxi }_{{\text{t}}} = 0.{1}$) and optimum parameters are shown in Tables 1, 2 and 3. Some equations were used in past studies^7,84–88^. For the optimum TMD, similar equations are also used by changing the negative stiffness damping system. Several iteration techniques for the optimum damping and tunning of TMNSDI are used based on the minimum mean square error. The three different optimization options for the optimum parameter of TMNSDI are given in the form of the subsequent expressions:

For minimization of $\tilde{\sigma }_{{x_{s} }}^{2}$ response

18 $$\upxi _{{\text{t}}}^{{{\text{opt}}}} = \sqrt {\frac{{\upmu \left( {4 + 2.5\upmu } \right)}}{{6\left( {1 +\upmu } \right)\left( {2 +\upmu } \right)}}} $$

19 $$ {\text{f}}^{{{\text{opt}}}} = \frac{2}{{1 +\upmu }}\left( {\frac{{\sqrt {1 +\upmu /2} }}{{1 +\upxi _{{\text{s}}} }}} \right) $$

20 $$ {\mathop{\sigma_{s,opt}}\limits^{\sim}}^{2} = \frac{{2\pi^{2} \xi_{s} }}{5\sqrt \mu }\left[ {1 - \frac{3}{7}\sqrt {\frac{{\xi_{s} }}{\sqrt \mu }} } \right] $$

For minimization of $\tilde{\sigma }_{{x_{{\mathop {x_{a} }\limits^{..} }} }}^{2}$ response

21 $$\upxi _{{\text{t}}}^{{{\text{opt}}}} = \sqrt {\frac{{\upmu \left( {6 + 15\upmu } \right)}}{{8\left( {1 +\upmu } \right)\left( {2 +\upmu } \right)}}} \left( {1 +\upxi _{{\text{s}}} } \right) $$

22 $$ {\text{f}}^{{{\text{opt}}}} = \sqrt {\left( {1 - \frac{\upmu }{2}} \right)} \left[ {1 + 2.5\upxi _{{\text{s}}}^{1.5} \left( {0.4 -\upmu ^{4} } \right)} \right] $$

23 $$ \tilde{\sigma }_{{x_{{\mathop {x_{a} }\limits^{..} ,opt}} }}^{2} = \frac{{4\xi_{s} }}{{\left( {1 + 4\xi_{s}^{2} } \right)\sqrt \mu }}\left[ {1 - \frac{7}{9}\sqrt {\frac{{\xi_{s} }}{\sqrt \mu }} } \right] $$

For maximization of EDI response

24 $$ {\text{f}}^{{{\text{opt}}}} = \frac{{\left( {1 + 4\sqrt\upmu \upxi _{{\text{s}}}^{2} } \right)}}{{\sqrt {1 +\upmu } }} $$

25 $$\upxi _{{\text{t}}}^{{{\text{opt}}}} = \left( {1 +\upxi _{{\text{s}}} } \right)\sqrt {\frac{{4\upmu }}{{8\left( {1 +\upmu } \right)\left( {1.5 -\upmu } \right)}}} $$

26 $$ {\text{EDI}}_{{{\text{opt}}}} = 1 - \frac{{5\upxi _{{\text{s}}} }}{{\sqrt {\left( {1 +\upmu } \right)\left( {\upmu +\upxi _{{\text{s}}} } \right)} }} $$

**Figure 2:**
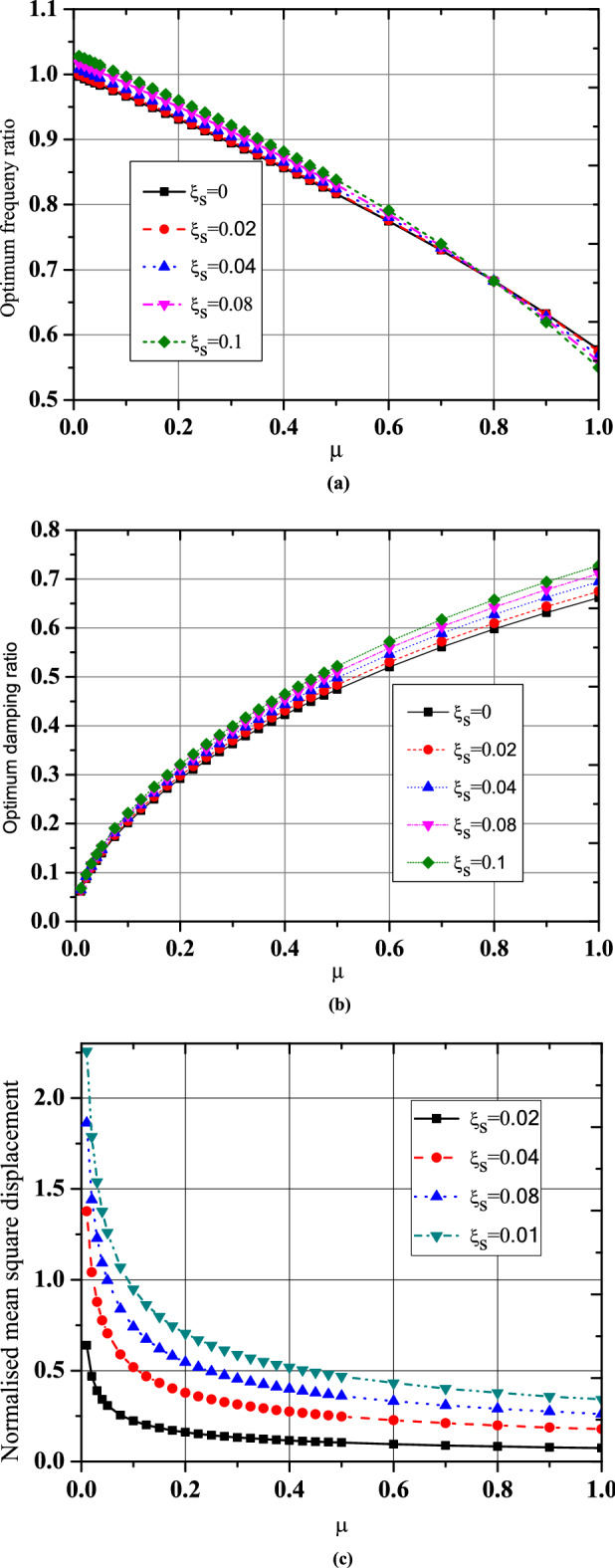
Minimization of displacement response variance: comparison of the results obtained using numerical search technique and explicit formulae.

**Figure 3:**
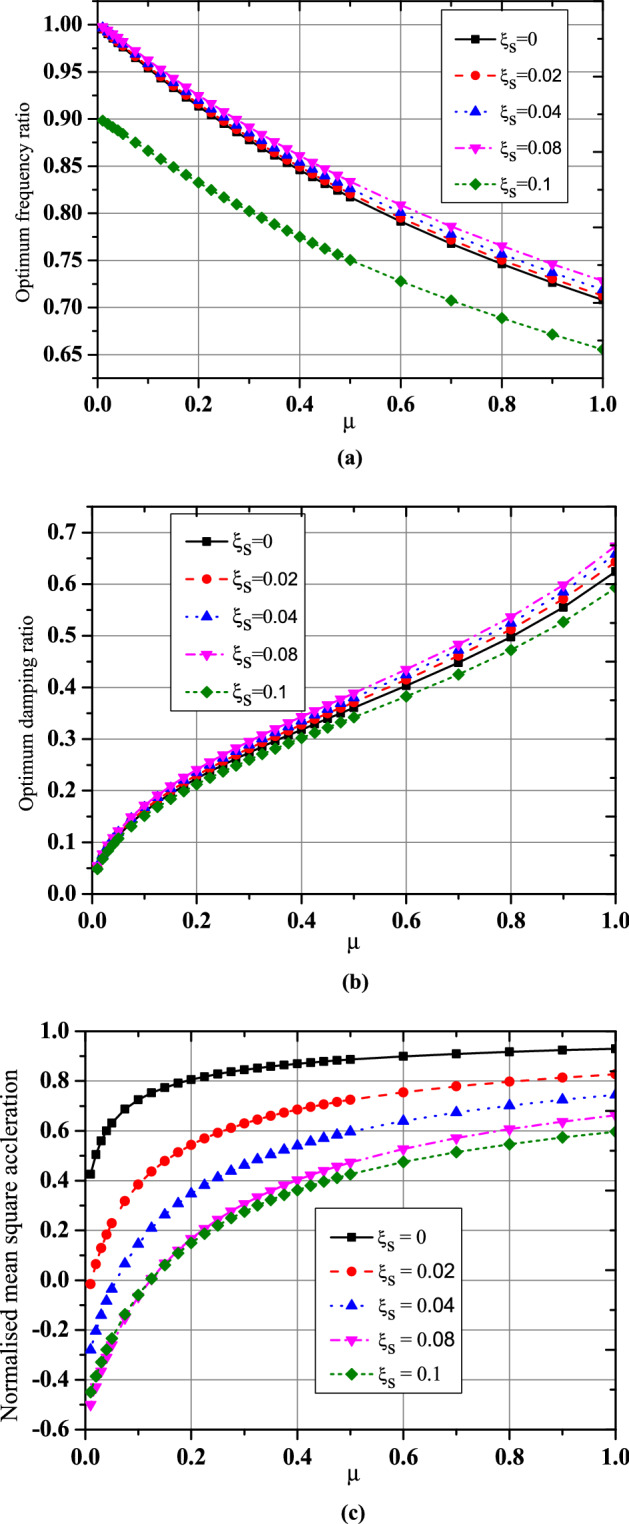
Minimization of acceleration response variance: comparison of results obtained using numerical search technique and explicit formulae.

**Figure 4:**
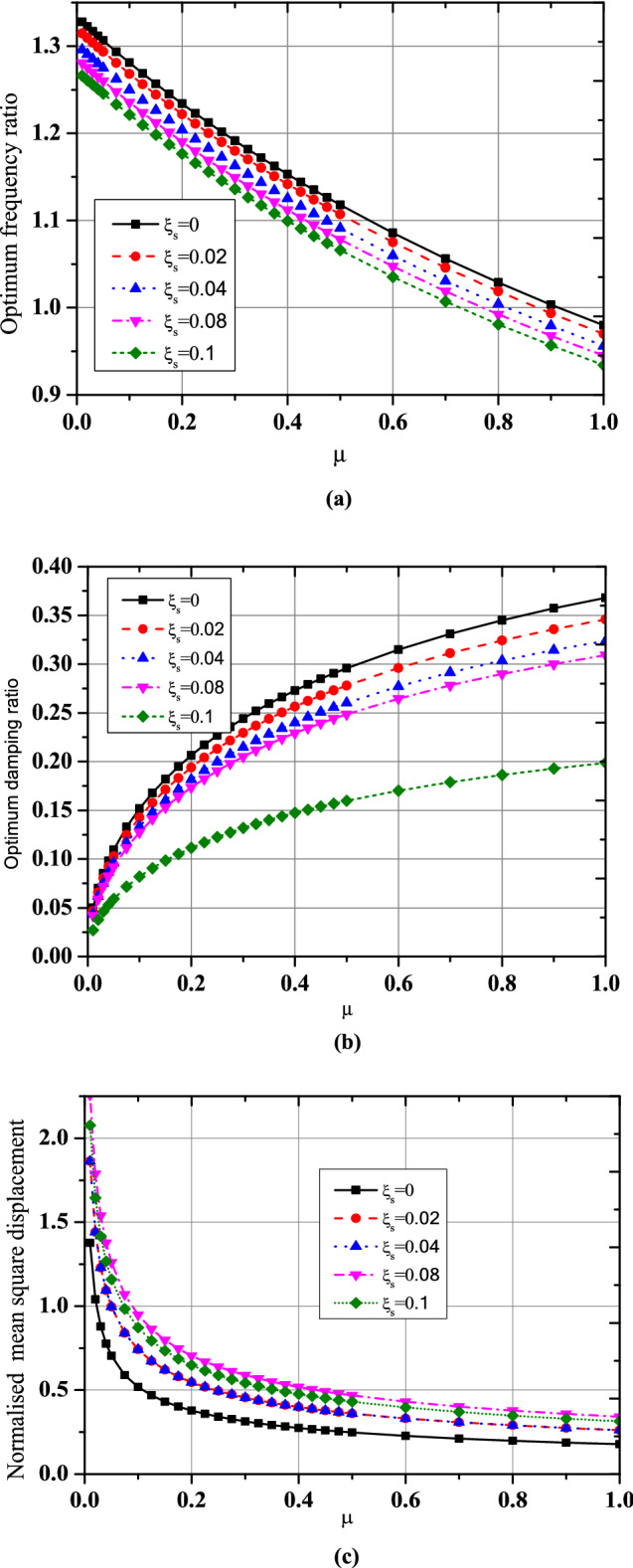
Maximization of EDI: comparison of the results obtained by numerical search technique and explicit formulae.

**Figure 5:**
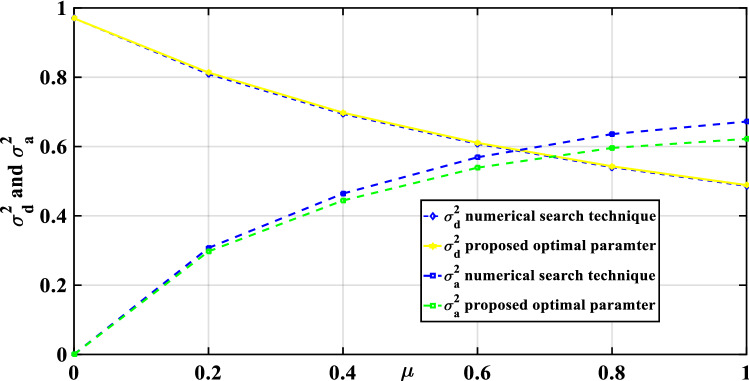
Numerical search technique and proposed empirical formulae of optimum parameters of base-isolated structure with TMNSDI comparisons.

For the three optimization cases (i.e., acceleration, displacement, and EDI), the numerical search approach and the developed closed form of expressions were compared for optimal TMNSDI. The predicted values of optimized TMNSDI and the values determined using numerical search techniques of the algorithm were compared for the autonomous variables of ${\upmu }$ and ${\upxi }_{{\text{s}}}$ for all three cases. The curve fitting technique and proposed explicit techniques show good agreement for optimal parameters of SDOF system with TMNSDI with deviations within the range of $\pm 5\%$. Figures 2, 3 and 4 show the comparisons of the optimal parameters for TMNSDI obtained using the numerical search technique and explicit formulae for minimization of displacement, minimization of acceleration, and maximization of EDI. From the numerical optimized values calculation, empirical equations were derived from the closed form of expressions. The results coincide with concentrations at the extreme explicit of ${\upmu }$-axis (${\upmu } = 0.0{2}$ and ${\upmu } = {1}.0{1}$) for the chosen structural damping. The optimum response of an SDOF system within the specified range of ${\upmu }$ and ${\upxi }_{{\text{s}}}$ empirical equations were obtained from the closed form of expressions for $\tilde{\sigma }_{{x_{s,} opt}}^{2}$, $\tilde{\sigma }_{{\ddot{x}_{a,} opt}}^{2}$ and EDI_opt_ and results coincide for extreme explicit expressions, as shown in Fig. 5.

## Isolation system modelling

### Ageing effects

**Figure 6:**
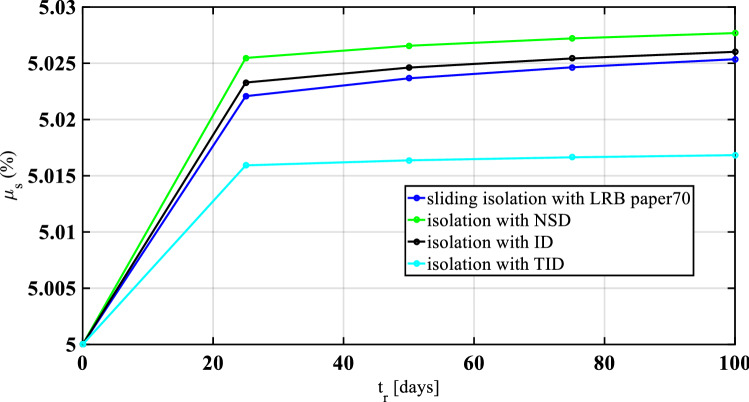
Aging effects on the performance of isolation systems.

In the event of an earthquake, base isolation of structures causes a reduction in fundamental vibration periods and increases the vertical and horizontal stiffnesses over a long time. Lower shear modulus, very high damping, substantially improper vulcanization, and rubber compound influence are dependent factors of ageing, as reported by previous researchers^89^. The following equations are considered for the aging effect on the static friction coefficient over time, considering the negative stiffness damper (NSD), inerter damper (ID), and tuned inerter damper (TID) are control devices.

27 $$ {\upmu }_{{\text{s,NSD}}} = {\upmu }_{{{\text{SO}}}} + {0}{\text{.0176t}}^{{{0}{\text{.1}}}} $$

28 $$ {\upmu }_{{\text{s,ID}}} = {\upmu }_{{{\text{SO}}}} + {0}{\text{.0196t}}^{{{0}{\text{.1}}}} $$

29 $$ {\upmu }_{{\text{s,TID}}} = {\upmu }_{{{\text{SO}}}} + {0}{\text{.0296t}}^{{{0}{\text{.1}}}} $$

where ${\upmu }_{{{\text{SO}}}}$ is the initial (unaged) value of the friction in the static condition, and *t* is the age measured in years. Figure 6 shows the variations of the aging effect of isolation with different control devices. The maximum static friction occurs for base isolation with NSD at an initial value of 5.025 and increases gradually up to 5.028 for 20 days and 100 days, respectively. The minimum static friction response occurs for base isolation with TID at an initial 5.015 and increases gradually up to 5.016 and 5.018 for 20 days and 100 days, respectively. The results trends show agreement with the experimental values reported by Mazza (2018) for base isolation using lead rubber bearings (LRB).

### Air temperature

**Figure 7:**
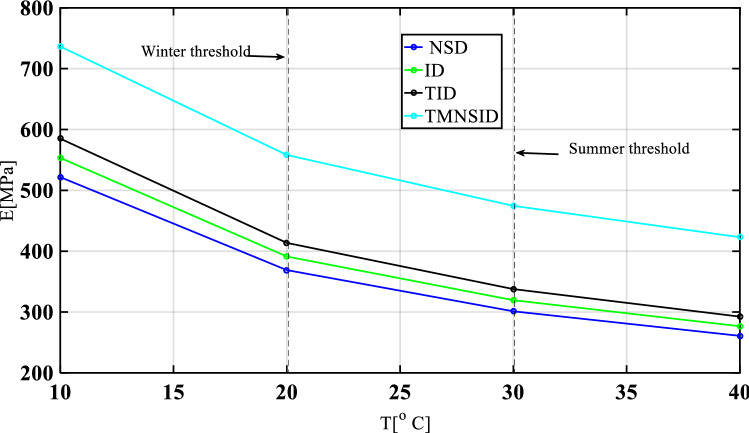
Performance of isolation systems due to air temperature under high damping isolation.

Base isolations with various control devices such as negative stiffness damper (NSD), inerter damper (ID), tuned inerter damper (TID), and TMNSDI were analyzed with damping properties. The increase in temperature decreases modulus of elasticity. Figure 7 shows the variation of modulus of elasticity with the air temperature. The maximum elastic modulus occurs for TMNSDI, and the minimum occurs for NSD at all air temperatures. At lower temperature, elastic modulus was higher and at higher temperature the elastic modulus decreased in all cases of isolation systems.

## Model verification

**Figure 8:**
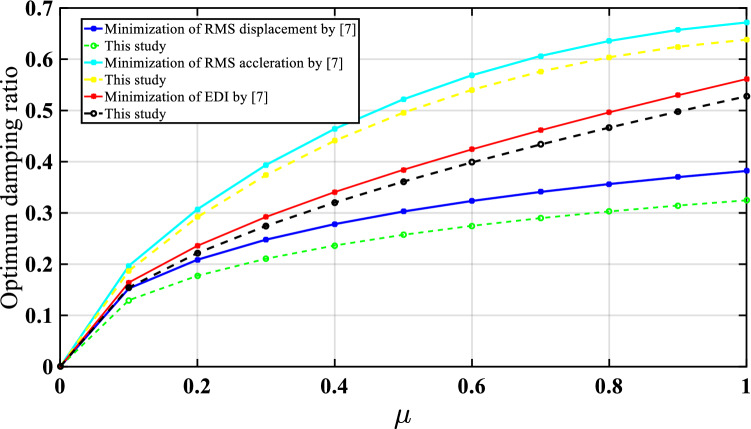
Optimum damping ratio of TID^7^ and TMNSDI (this study) numerical verification of base isolated structure with control devices.

**Figure 9:**
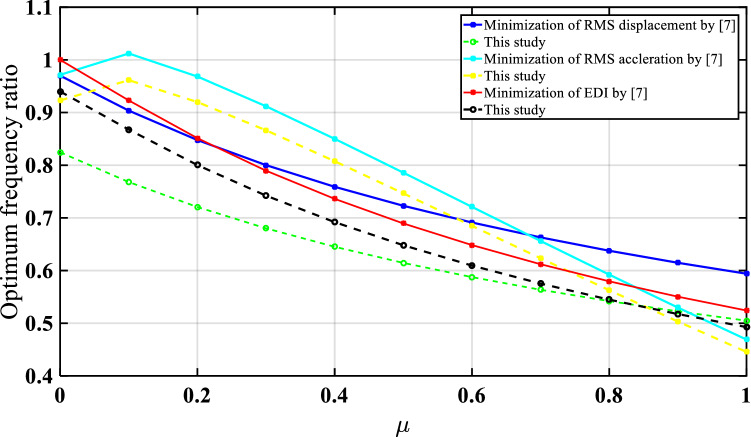
Optimum tunning frequency ratio of TID^7^ and TMNSDI (this study) numerical verification of base-isolated structure with control devices.

In this study, the base-isolated five-story structure was exposed to seismic load under Gaussian white noise excitation, the response was controlled by using tuned inerter damper (TID) and TMNSID, and the model was validated using the results obtained by^7^. The analysis was carried out using MATLAB^90^. The variations of results with damping and optimum frequency ratios for different inertia ratios show good agreement with that reported by^7^, as shown in Figs. 8 and 9, respectively. It is observed that the trend of results is similar to that reported by the previous researchers.

## Response of excitations due to pulse and real earthquake

### Response of pulse type of earthquake

**Figure 10:**
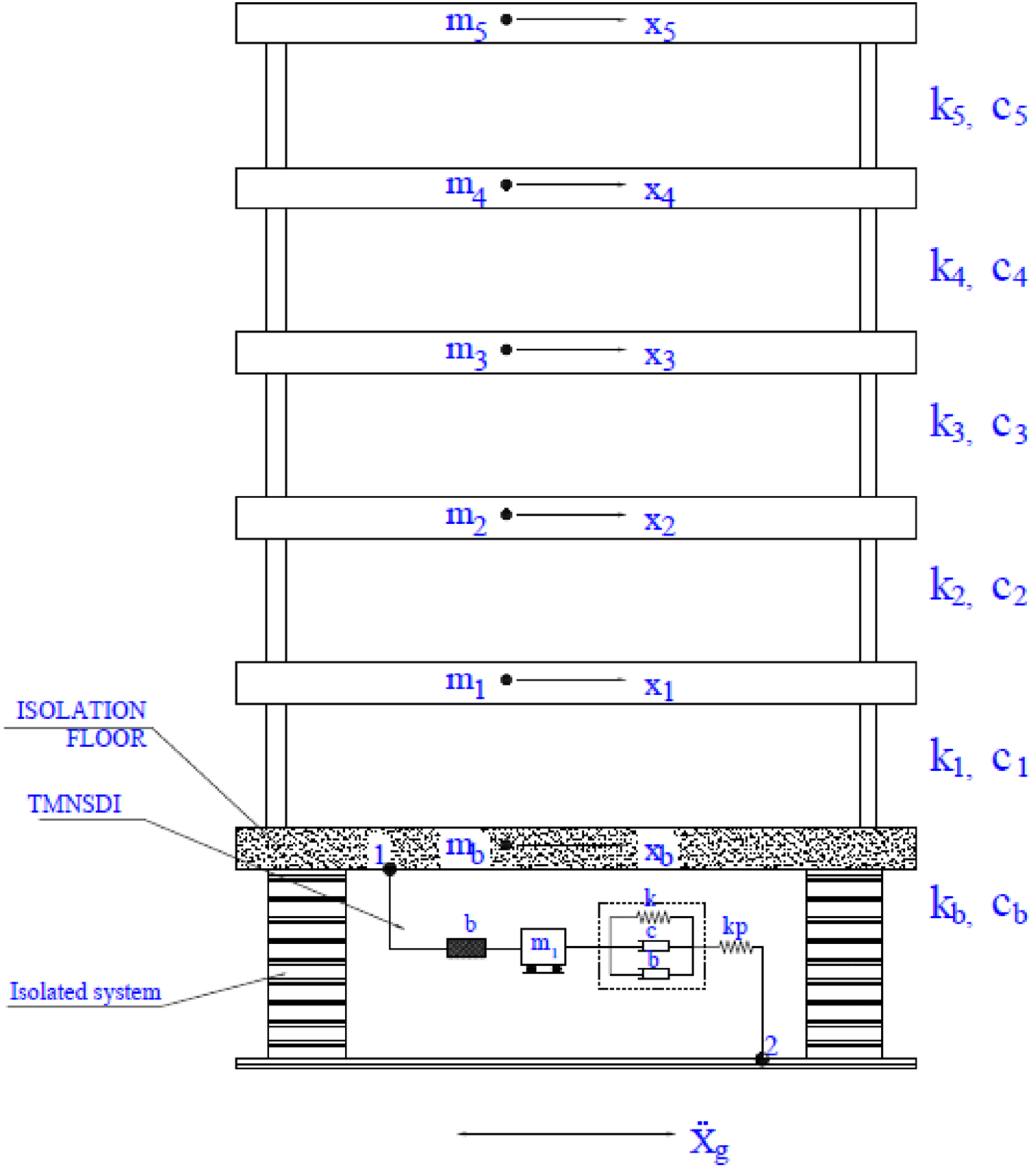
Flexible base-isolated structure supplemented with TMNSDI for the structural model.

Figure 10 shows the model of the flexible base-isolated structure with TMNSDI. The parameters considered for the five-story base-isolated structure (adopted from^91^) included m_1_, m_2_, m_3_, m_4_, m_5,_ and m_b_ (mass of all floors and base raft locations) and k_1,_ k_2_, k_3_, k_4,_ and k_5_ (stiffness of all floors). The magnitudes of stiffness were considered as follows: k_1_ = 15 k, k_2_ = 14 k, k_3_ = 12 k, k_4_ = 9 k, and k_5_ = 5 k. The fundamental natural period of 2 s was considered with the natural frequency of stiffness. With a fixed base, the five natural frequencies of the structure were considered as 15.71, 38.48, 60.84, 83.12 and 105.37 rad/s, respectively, for all five floors.

The governing equation of motion of structure with TMNSDI is as follows:

30 $$ {\text{M}}{\ddot{\text{x}}}(\text{t}) + \text{C}{\dot{\text{x}}}(\text{t}) + \text{Kx}(\text{t}) + \text{DF}_{{\text{s}}} {\text{(t)}} = - {\text{E}}{\mathop{{\text{x}}_{{\text{g}}}}\limits^{..}} {\text{(t)}} $$

where C M, and K are damping, the mass, and stiffness matrices of the base-isolated structure, respectively; x(t) denotes lateral displacement (relative to ground) vector at time t; D represents location matrix for the vector of control forces F_s_(t) produced by inertial devices, E is the vector containing the vibrating masses and ${\mathop{{\text{x}}_{{\text{g}}}}\limits^{..}} {\text{(t)}}$ are the earthquake ground acceleration.

## Response of real type of earthquake

Table 4 shows eight different real ground motion data considered for analysis of base-isolated structure, as reported by PEER^92^. The importance of nonstationary excitation on the base-isolated structure is also considered^93^.

**Table 4:** Properties of earthquake ground motion data.

Earthquake	Station	Peak ground acceleration (PGA)	Magnitude (M_w_)	Component	Fault
Bam Iran (26–12–2003)	Bam	0.348	6.6	EW	FF
Darfield New Zealand (9–03–2013)	Darfield	0.2	7.1	NS	FF
Darcy turkey (11–12–1999)	Izmit	0.45	7.4	EW	NF
El Mayor Cucapah (4–4–2010)	Baja California	0.52	7.2	NS	FF
Park field (28–09–2004)	California	0.55	4.7	EW	FF
Tottori Japan (06–10–2000)	Western Honshu	0.23	6.6	NS	FF
El Centro (19–05–1940)	Terminal Substation	0.19	6.9	EW	NF
Kobe (17–01–1995)	Great Hanshin	0.18	6.9	NS	FF
Koyna (11–12–1967)	Terminal Substation	0.25	6.6	NS	FF
Mexico (19–09–1985)	Mexico	0.22	8.1	EW	FF

## Fragility analysis for base-isolated structure with ground motion data

**Figure 11:**
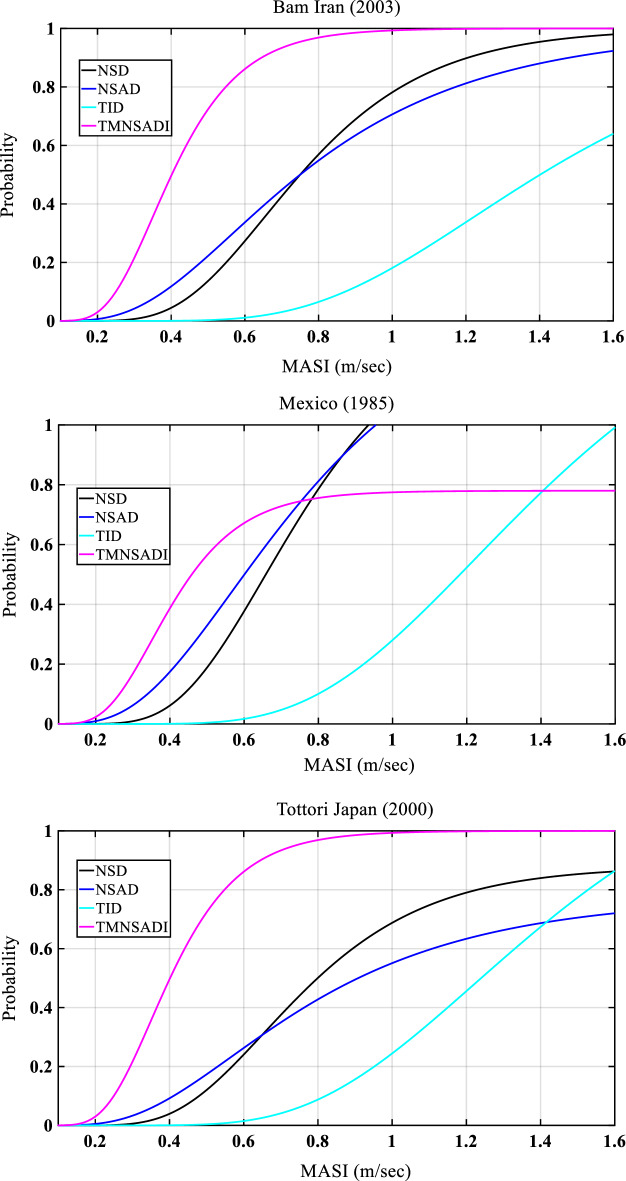
Fragility curves of the structural systems with various control devices for base-isolated structure with TMNSDI under pulse type of seismic load.

**Figure 12:**
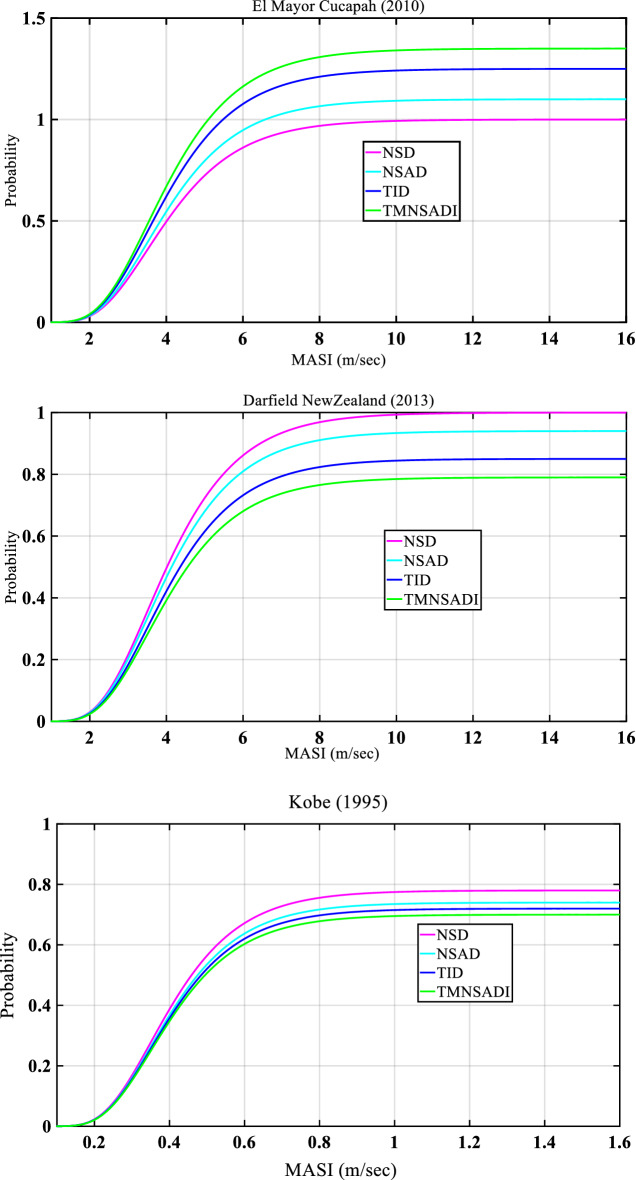
Fragility curves of the structural systems with various control devices for base-isolated structure with TMNSDI under white noise excitation.

Figures 11 and 12 show the results of the fragility analysis of the structure under seismic load and white noise excitations for different incidences of earthquake occored in the past (Bam Iran 2003, Mexico 1985, Tottori Japan 2000, El Mayor Cucapah 2010, Darfield New Zealand 2013, and Kobe 1995). The fragility curve is defined as a curve obtained from probability or demand versus modified acceleration spectrum intensity (MASI). The curve represents the reductions in the probability response from various control devices such as negative stiffness damper, negative stiffness amplifying damper, tunned inertia damper, and tuned negative mass stiffness amplifying damping inertia. The TID damper controls the peak response for Bam Iran (2003) ground motion data. In the case of a base-isolated structure with TMNSDI under white noise excitation, the fragility curve works as a vital role in the reduction of the response.

**Figure 13:**
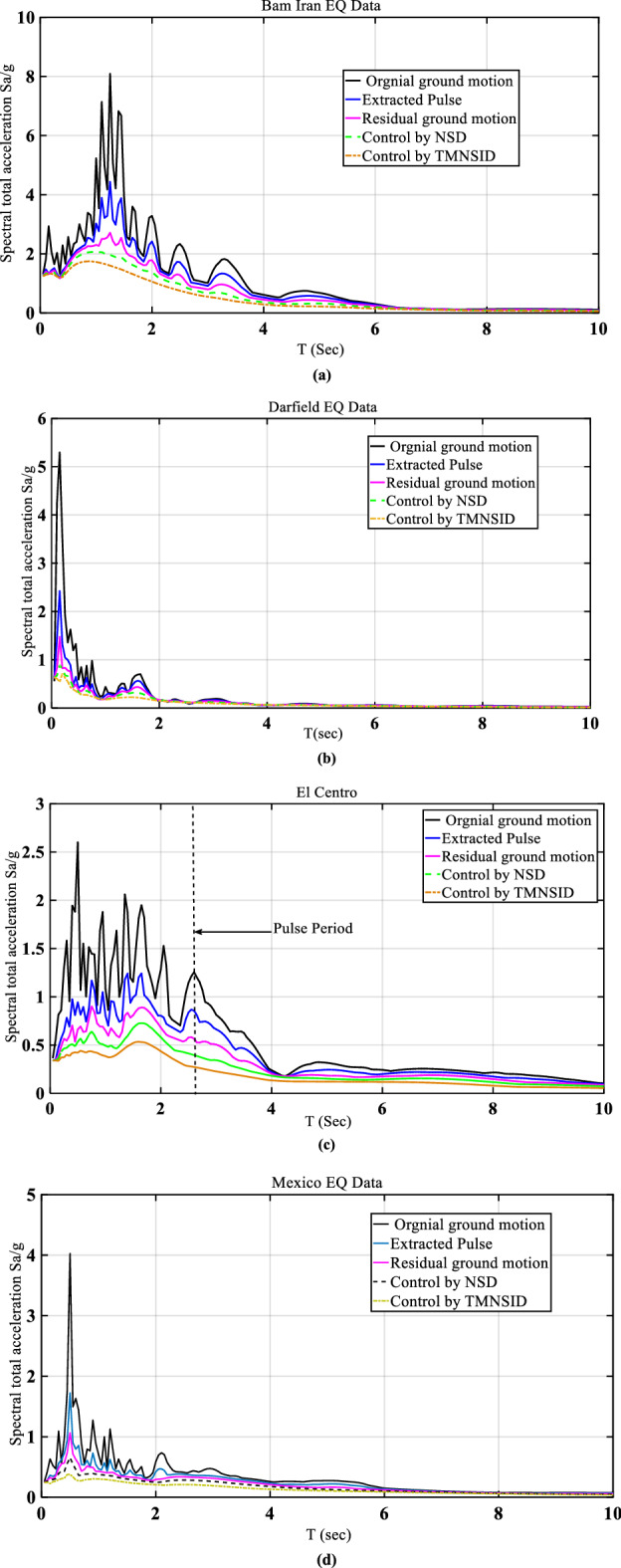
Control of total spectral acceleration by various devices.

**Figure 14:**
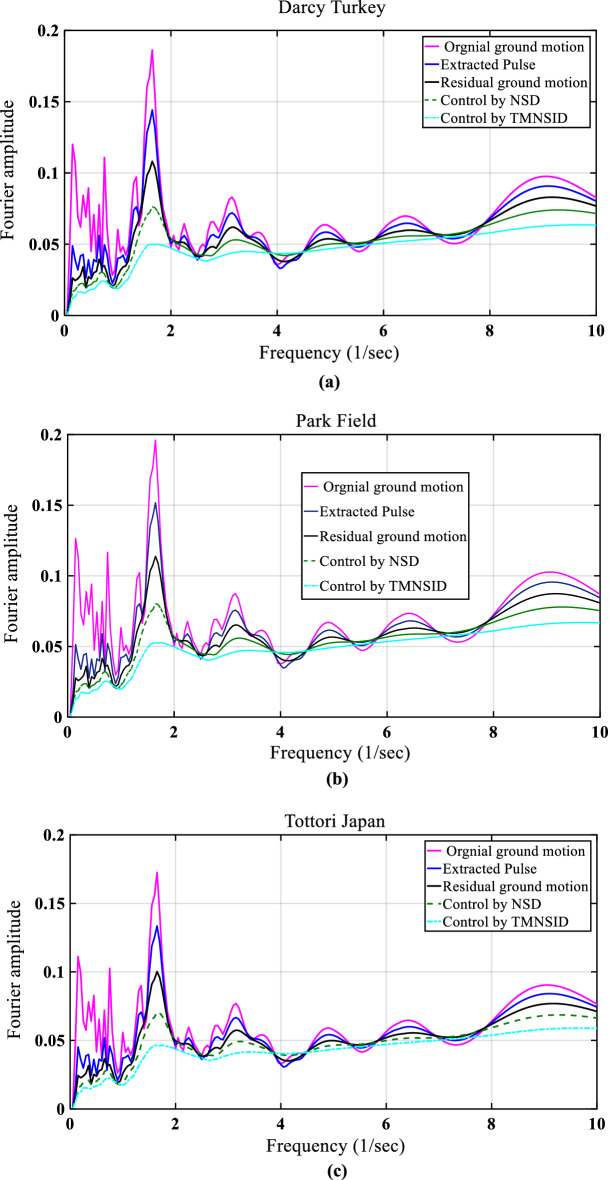
Fourier spectra for near-fault earthquakes response reduction by control devices.

**Figure 15:**
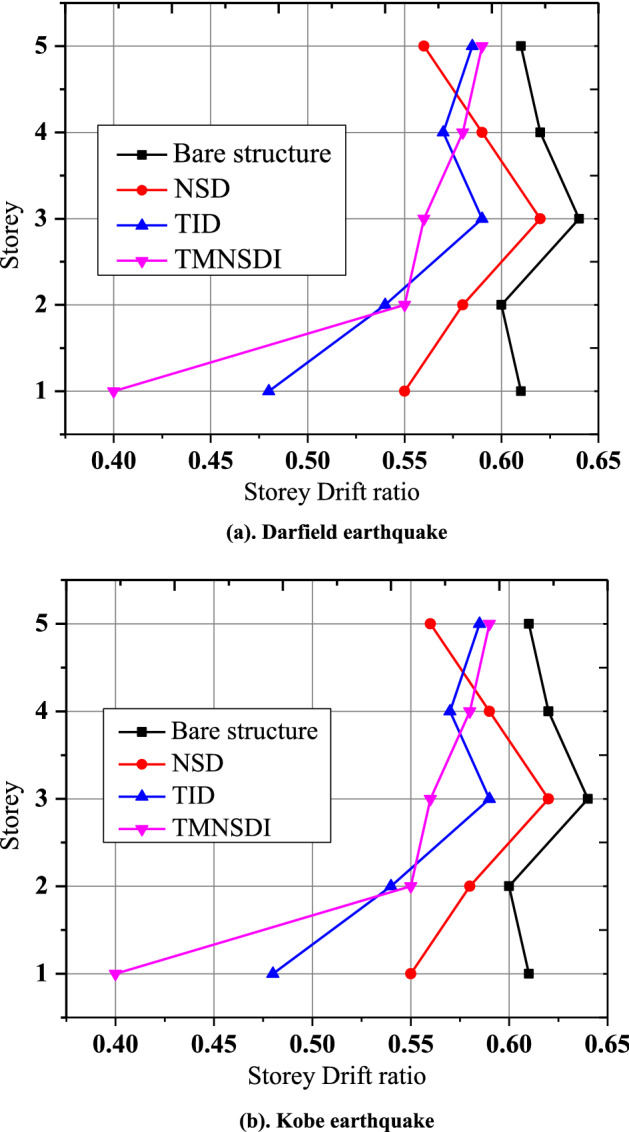
Control of inter-story drift ratio by various devices.

Figure 13 shows the control of spectral accelerations by using various control devices. The maximum spectrum accelerations correspond to Bam's ground motion, and the minimum accelerations correspond to Mexico ground motion. The maximum spectral accelerations are controlled by using TMNSDI compared to other devices. Figure 14 shows the control of Fourier amplitude response reductions for different ground motion data. The maximum amplitude occurs for Darcy turkey ground motion with an amplitude of 0.19 and is controlled at 0.04 amplitude with TMNSDI. Figure 15 shows control of the inter-story displacement of the base-isolated structure with various control devices. The maximum inter-story displacement is controlled by using TMNSDI compared with other devices. The maximum inter-story displacement occurs on the first floor, and the minimum inter-story displacement occurs on the fifth floor. In the case of Darkfield, ground motion for base-isolated structure with TMNSDI, the story drift ratio is reduced by 70%, and in the case of Kobe, ground motion for base-isolated structure with TMNSDI, the story drift ratio is reduced by 75%.

**Figure 16:**
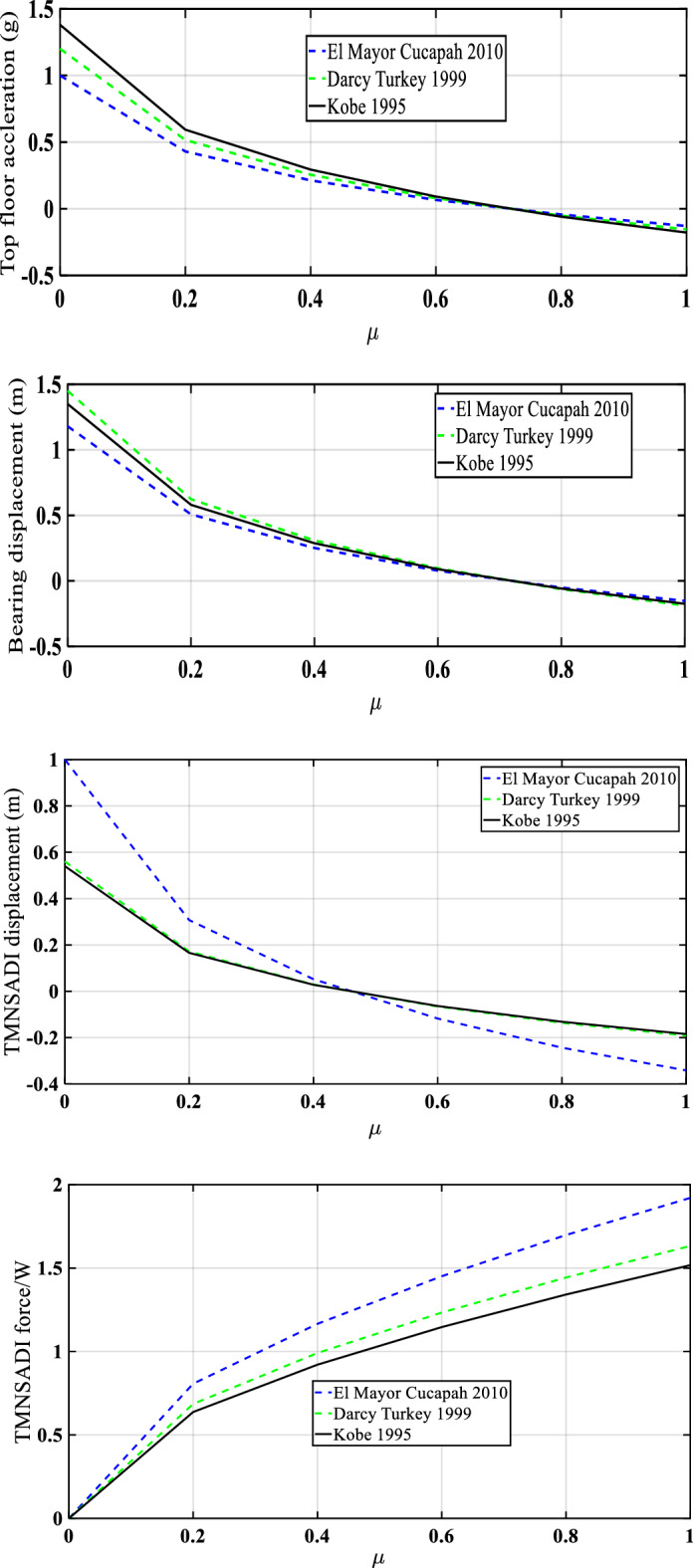
Five-storey base-isolated structure with TMNSDI on peak top floor absolute acceleration, relative base displacement, the relative displacement of TMNSDI, and base shear force due to inertance of TMNSDI influence (T_b_ = 3 s).

**Figure 17:**
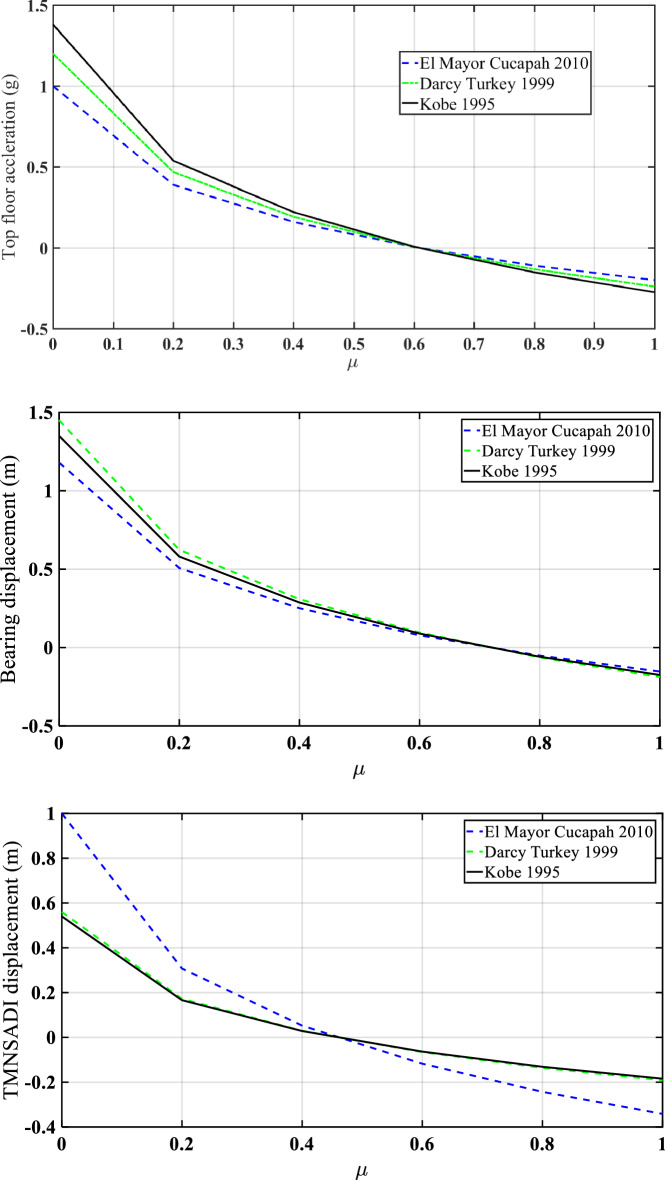
Five-storey base-isolated structure with TMNSDI on peak top floor absolute acceleration, relative displacement of TMNSDI and base shear force due to inertance of TMNSDI influence (T_b_ = 2 s) under seismic pulse type load.

Figures 16 and 17 show the response reductions of a five-story structure under real and pulse types of earthquakes in terms of displacement, bearing displacement, and the ratio of force by weight, using various control devices. The maximum reduction in the response is by using TMNSDI. The three different ground motion data considered are: El Mayor Cucapah 2010, Darcy Turkey 1999, and Kobe 1995. The maximum top floor acceleration occurs at El Mayor Cucapah 2010 ground motion as the inertance ratio increases. Top-floor acceleration initially increases and then decreases. Kobe 1995 has minimum top-floor acceleration with respect to inertance ratio. Maximum bearing displacement response was in case of Darcy Turkey 1999, and minimum ground motion response was in case of El Mayor Cucapah 2010.

Figure 17 represents the five-story base-isolated structure with TMDSDI under pulse-type seismic load analyzed for three different ground motions consisting of the same natural frequency variations with respect to different mass ratios. As the mass ratio increases, normalized force increases. Initially, it is zero and reaches a maximum for the corresponding inertance ratio of one. The same natural frequencies are in cases of El Mayor Cucapah 2010, Darcy Turkey 1999, and Kobe 1995 having different ground motions. The maximum normalized force occurred for El Mayor Cucapah 2010 having ground motion of isolated base structure under TMNSDI. The minimum normalised force was found for Kobe 1995 ground motion data.

## Conclusions

In the present work, a hybrid control device, TMNSDI, capable of controlling the response of the structure under real ground motion data, is proposed. Base-isolated structures with TMNSDI under real ground motion data and pulse-type earthquake load investigation were carried out. Stationery and filter white noise excitation of optimum damping and tunning frequency ratio of TMNSDI were obtained by using a numerical search technique. For practical applications, the conventical method was used for curve fitting, tunning frequency, and explicit formulae of TMNSDI. The following conclusions are drawn based on the results:

1. Optimum parameters of TMNSDI with base-isolated in terms of displacement and acceleration are controlled effectively. The response is almost stationary for a mass ratio above 0.4.
2. Optimum damping ratio, frequency ratio, and mass ratio were obtained using the numerical searching technique considering the derived explicit expressions for TMNSDI parameters.
3. Fragility curve results show a 40% reduction of displacement and acceleration.
4. The spectrum acceleration response reduction obtained using real ground motion data indicates a maximum reduction for TMNSDI.
5. The spectrum amplitude response reduction using real ground motion data indicates a maximum reduction for TMNSDI.
6. Story drift ratio results show a 70% reduction in Darfiled ground motion, whereas there is a 73% reduction in El Kobe ground motion for base-isolated structure with tuned mass negative stiffness damper inerter.

## Data Availability

The datasets generated during and/or analysed during the current study are available from the corresponding author on reasonable request.

## Appendix A

### A1 Provenance of fragility anxiety

Aleatory and epistemic are the multiple sources of excitation for the uncertainty of an inherent fragility^94,95^.

1. Natural randomness of ground motion excitation for the relationship of EDP-IM (Engineering Demand Parameter-Intensity Measure) obtained from the record-to-record inconsistency. Hence less record of ground motion results in the same level of assurance in the approximation of distribution for EDP-IM.
2. For computation efficiencies, typically in case of uncertainty model^96^, flawed modelling capabilities, especially considering simplified models, are mentioned.
3. Strength, ductility, mass, or stiffness are the different model parameters showing uncertainty due to incomplete information are discussed in the literature^97–99^.
4. The deficient procedure-related uncertainty such as evil regression; a deficient IM; or a scarce investigation method, for example, by means of nonlinear static investigation for a high-rise structure should be avoided as much as possible.

### A2 Contextual of fragility

The idea of intensity measurement is the notion of fragility intimately tied to the site for the interface between the structural engineer and seismology of measurement of severity and intensity measure. A hazard curve can be obtained for a scalar variable of IM as a scalar variable, typically peak ground motion. Herein, the opinion of the presentation-founded valuation problem, seismologists model any faults causation earthquakes that might influence the site below study and review all data into a single hazard curve [or hazard surface in the case of a vector^100^ on behalf of the unkind annual frequency (MAF), λ, of certain exceptional levels of seismic intensity.

### A3 Algorithm constituent-level seismic fragility assessment algorithm

1. For every IM level
2. For every story (or component) i and story-level (or component-level) capacity
3. Estimate $F_{LS}^{k}$ via Eq. (31)
4. End for
5. End for
6. Optionally, combine $F_{LS}^{k} \left( {IM} \right)$ to obtain $F_{LS} \left( {IM} \right)$ via (32)
  31 $$ F_{LS}^{k} = \frac{{\sum\limits_{j = 1}^{{N_{\sec } }} {I\left[ {any(EDP_{i}^{j} > EDP_{i,C}^{k} )\left| {IM} \right|} \right]} }}{{N_{rec} }} $$
  32 $$ F_{LS} = \frac{{\sum\nolimits_{k = 1}^{{N_{rec} }} {I\left[ {any(EDP_{i}^{j} > EDP_{i,C}^{k} )\left| {IM} \right|} \right]} \sum\nolimits_{k = 1}^{{N_{c} }} {I\left[ {any(EDP_{i}^{j} > EDP_{i,C}^{k} )\left| {IM} \right|} \right]} }}{{N_{c} N_{rec} }} $$
